# Antibody signatures of asymptomatic *Plasmodium falciparum* malaria infections measured from dried blood spots

**DOI:** 10.1186/s12936-021-03915-8

**Published:** 2021-09-23

**Authors:** Christine F. Markwalter, Myat Htut Nyunt, Zay Yar Han, Ricardo Henao, Aarti Jain, Omid Taghavian, Philip L. Felgner, Kay Thwe Han, Myaing M. Nyunt, Christopher V. Plowe

**Affiliations:** 1 Duke Global Health Institute, Duke University, Durham, NC USA; 2 Department of Medical Research, Myanmar Ministry of Health and Sports, Yangon, Myanmar; 3 Department of Biostatistics and Bioinformatics, Duke University, Durham, NC USA; 4 Vaccine Research & Development Center, School of Medicine, University of California Irvine, Irvine, CA USA; 5 University of Maryland School of Medicine, Baltimore, MD USA

**Keywords:** Serology, Antibody responses, Protein microarrays, Asymptomatic malaria

## Abstract

**Background:**

Screening malaria-specific antibody responses on protein microarrays can help identify immune factors that mediate protection against malaria infection, disease, and transmission, as well as markers of past exposure to both malaria parasites and mosquito vectors. Most malaria protein microarray work has used serum as the sample matrix, requiring prompt laboratory processing and a continuous cold chain, thus limiting applications in remote locations. Dried blood spots (DBS) pose minimal biohazard, do not require immediate laboratory processing, and are stable at room temperature for transport, making them potentially superior alternatives to serum. The goals of this study were to assess the viability of DBS as a source for antibody profiling and to use DBS to identify serological signatures of low-density *Plasmodium falciparum* infections in malaria-endemic regions of Myanmar.

**Methods:**

Matched DBS and serum samples from a cross-sectional study in Ingapu Township, Myanmar were probed on protein microarrays populated with *P. falciparum* antigen fragments. Signal and trends in both sample matrices were compared. A case-control study was then performed using banked DBS samples from malaria-endemic regions of Myanmar, and a regularized logistic regression model was used to identify antibody signatures of ultrasensitive PCR-positive *P. falciparum* infections.

**Results:**

Approximately 30% of serum IgG activity was recovered from DBS. Despite this loss of antibody activity, antigen and population trends were well-matched between the two sample matrices. Responses to 18 protein fragments were associated with the odds of asymptomatic *P. falciparum* infection, albeit with modest diagnostic characteristics (sensitivity 58%, specificity 85%, negative predictive value 88%, and positive predictive value 52%).

**Conclusions:**

Malaria-specific antibody responses can be reliably detected, quantified, and analysed from DBS, opening the door to serological studies in populations where serum collection, transport, and storage would otherwise be impossible. While test characteristics of antibody signatures were insufficient for individual diagnosis, serological testing may be useful for identifying exposure to asymptomatic, low-density malaria infections, particularly if sero-surveillance strategies target individuals with low previous exposure as sentinels for population exposure.

**Supplementary Information:**

The online version contains supplementary material available at 10.1186/s12936-021-03915-8.

## Background

Over the last decade, Myanmar, the country in the Greater Mekong Subregion that historically has had the highest burden of malaria, has made considerable progress toward malaria elimination. Since 2010, the number of malaria cases in Myanmar has decreased by more than 94% [1], corresponding to increased malaria control and treatment efforts [2]. This progress has presented new challenges for malaria control and elimination programmes to target interventions effectively and allocate limited resources to optimize impact on malaria transmission. These challenges include heterogeneous and focal transmission in geographic “hotspots” and population “hotpops” [3, 4] as well as low-density asymptomatic infections, which are thought to be a silent reservoir of malaria transmission and represent the majority of infections in low-transmission settings [5–7]. Ultrasensitive PCR (usPCR) methods have revealed that in areas of Myanmar, an estimated 85–99.5% of infections are asymptomatic [2, 8–10], making them potentially important targets for malaria elimination. While usPCR has proven valuable for characterizing malaria distribution in Myanmar, it is not optimal for long-term surveillance, as it cannot be performed in the field and has high equipment and reagent costs [11]. These challenges are exacerbated in elimination zones, where low transmission levels mean that large sample sizes are required for precise prevalence estimates [12].

Antibody biomarkers represent promising tools for malaria surveillance in elimination settings that may mitigate some of the challenges of measuring parasite prevalence with usPCR. Antibody responses can reveal past exposure in addition to current infection status, adding substantially more information to population-level measures of transmission [13, 14]. Antibody biomarkers are also readily detectable in field-friendly diagnostic formats similar to commercially available lateral flow-based rapid diagnostic tests (RDTs), and have been targeted in point-of-contact tests for other infectious pathogens, such as HIV [15] and the hepatitis C virus [16]. Additionally, it is likely that in individuals with low-density infections, malaria-specific antibodies are more readily detectable than parasite-specific antigens currently detected by currently available RDTs.

Protein microarrays are useful for screening antibody responses against large panels of antigens and protein fragments to identify potential biomarkers of exposure. In this format, antigens are printed onto nitrocellulose pads, antibodies in samples bind to antigens, and fluorescently-labelled secondary antibodies enable quantitative readout of signal. Protein microarrays have been used extensively to screen malaria-specific antibody responses, including those that elicit protection from uncomplicated and severe disease [17–20] and transmission [21, 22], as well as for markers of exposure [14, 23–25]. In most studies, the sample matrix probed on protein microarrays has been human serum, the gold standard for serological studies. However, the complexity of sample processing and cold chain requirement for collecting and transporting serum is a major limitation in hard-to-reach places where malaria is prevalent. Dried blood spots (DBS) are potentially useful alternatives to serum; they are simple to collect, durable, easy to transport, and present minimal biohazard risk. In this study, the performance of DBS was directly compared to that of serum in a matched, head-to-head study using protein microarrays populated with antigen fragments from the *Plasmodium falciparum* 3D7 reference genome (Pf250) [26]. It was also hypothesized that long-term exposure to low-density infections, which have been shown to persist for months [27], may elicit antibody signatures that could serve as biomarkers of infection. This hypothesis was tested by probing DBS on protein microarrays to identify antibody responses as markers for usPCR-positive (usPCR+) *P. falciparum* malaria infections.

## Methods

### Study settings and participants

A cross-sectional study was conducted in Ingapu Township, Myanmar, during the rainy season in August 2018. This region was previously characterized by the Myanmar National Malaria Control Programme as a medium-transmission area, with reported usPCR prevalence of 8.7% *P. falciparum* infections and 5.6% *Plasmodium vivax* infections during the rainy season in 2015 [11]. A study carried out in 2015 found the annual parasite index (API) in the region to be 1–5 malaria cases per 1000 individuals under surveillance per year [2]. Individuals aged 6 months or older, including pregnant women and the elderly, were enrolled in the study after provision of written informed consent. Parents/guardians provided written consent for participants younger than 18 years old. Written assent was also provided by children age 8 to 17 years of age. Enrollment in the study was offered to individuals attending a mobile clinic staffed by local public health volunteers. One hundred individuals were enrolled.

To identify potential markers of asymptomatic usPCR+ malaria infections, a retrospective case-control study nested within a previously reported 2015 cross-sectional survey [11] in which participants provided consent for additional analysis of samples was performed. Samples originated from Injanyang and Ann Townships, both of which have reported API > 5 cases per 1000 individuals under surveillance per year [2]. No participants reported malaria symptoms, nor were any RDT-positive. Antibody responses were measured from archived case and control DBS samples on Protein Saver 903 Cards. Cases (n = 50) were individuals who tested positive for *P. falciparum* by usPCR, and controls (n = 175) were individuals who tested negative for *P. falciparum* by usPCR. This study was designed specifically to identify antibody markers of usPCR+ *P. falciparum* malaria infections, so individuals with *P. vivax* mixed and mono-infections were included in the case and control groups, respectively, to account for potential cross-reactive responses. The case group included 25 individuals with mixed *P. falciparum* and *P. vivax* infections. The control group included 25 individuals who were *P. vivax*-positive.

### Sample collection

For the study comparing antibody responses from DBS and serum samples, capillary whole blood was collected by finger prick. Each individual was screened for malaria by RDT (SD Bioline Ag Pf/Pv). DBS samples (50 µL obtained by digital puncture) were collected on Whatman 3MM chromatography paper (WHA3030861) for usPCR analysis as previously described [11] and Protein Saver 903 Cards (WHA10534612) for analysis on protein microarrays. DBS were air-dried, placed in sealed plastic bags with desiccant, and stored at −80 °C for up to 7 months before analysis. Capillary whole blood was collected in 300 µL tubes, transported to the laboratory, centrifuged to isolate serum, and stored at −80 °C until protein microarray analysis.

### Ultrasensitive PCR (usPCR)

All samples were tested for *P. falciparum* and *P. vivax* infections by usPCR, which targets the parasite 18S rRNA gene and transcripts. Human actin DNA was used as an internal extraction control. Extraction and reverse transcription PCR (RT-PCR) were performed as previously described [11]. Briefly, nucleic acids were extracted from Whatman 3MM samples (50 µL) using a guanidine thiocyanate-isopropanol buffer spiked with β-mercaptoethanol. Nucleic acids were isolated, washed and eluted into 50 µL of TE buffer using Nunc glass fiber filter plates (VWR 73520-504). Extracts were stored at −80 °C. RT-PCR was performed in duplicate as previously described: reaction mixtures of 5 µL 2x Quantitect Multiplex Mastermix, 0.1 µL Quantitect Reverse Transcriptase Mix, 0.2 µL 1U/µL heat-labile uriacil-DNA glycosolase, 1.5 µL nucleic acid template, and 3.2 µL RNAse-free water containing primers and probes listed in Adams et al. were amplified following the same cycling conditions on a Roche Lightcycler 96 [28]. All data were analysed with LC96 software version 1.1. Samples were considered PCR-positive when duplicate measurements had Cq values below 37. Previously reported detection limits for this extraction and PCR protocol were 0.016 parasites/µL for *P. falciparum* and 49.5 copies/µL of 18 S rRNA for *P. vivax* [11].

### Elution of antibodies from DBS

Protein Saver 903 DBS samples were punched with a 6.35 mm hole punch (Staples 10573-CC) into 2-mL microcentrifuge tubes. Next, 150 µL Pierce Casein Blocking Buffer in PBS (Thermo Scientific 37528) with 0.3% Tween-20 was added to each tube, and samples were incubated overnight with gentle rocking at 4 °C. The following morning, DBS were removed and discarded, and 150 µL glycerol was added to stabilize the remaining supernatant. Eluates were stored at − 20 °C.

### Protein microarray construction

Microarrays were constructed as previously described [29]. Briefly, exons from *P. falciparum* (strain 3D7) were cloned and expressed in individual T7 *Escherichia coli*-derived in vitro transcription/translation (IVTT) reactions (GenScript). Reaction products, along with no-template IVTT control reaction mixtures, were printed directly onto nitrocellulose pads on glass slides (GraceBio).

### Protein microarray probing

Pf250 protein microarrays were probed as previously described [29]. Serum samples were diluted 1:100 in a solution of 20% *E. coli* lysate (GenScript) in GVS FAST Blocking Buffer (#10485356) and incubated at room temperature for 30 min. DBS eluates (150 µL) were combined with 50 µL 80% *E. coli* lysate in GVS Fast Blocking buffer for 30 min at room temperature. This dilution of DBS eluates approximates a 1:100 serum dilution. Matched DBS and serum samples were probed on the same slides to avoid batch effects. For the case-control study, malaria cases and controls were randomized on the microarray slides prior to probing to avoid batch effects. Protein microarrays were assembled into GraceBio Proplates (#246890), and the nitrocellulose microarray pads were rehydrated with 100 µL GVS FAST Blocking buffer for 30 min on an orbital shaker. Supernatants were aspirated before 100 µL of prepared DBS or serum samples were added to the pads. Samples were incubated on the protein arrays at 4 °C overnight with gentle rocking. The following morning, supernatants were aspirated and washed quickly 3× with TRIS Buffered Saline with 0.05% Tween-20 (TTBS) followed by 35-min TTBS washes on an orbital shaker. Biotin-conjugated goat anti-human IgG secondary antibody (Jackson Immunoresearch, 109-065-098) was diluted 1:400 and incubated on the pads (100 µL) for 1 h at room temperature on an orbital shaker. Pads were washed with TTBS as described above, and 100 µL of streptavidin-coated quantum dots (QDot 655 streptavidin, ThermoFisher Q10121MP) at 1:200 dilution in GVS FAST Blocking buffer was added to each pad and incubated for 1 h on an orbital shaker. Finally, microarrays were washed with TTBS as described above, rinsed in DI water, and air-dried by centrifugation for 5 min (1000*g*). Fluorescence intensities were measured using an ArrayCAM Imaging System from Grace BioLabs (Bend, Oregon).

### Data analysis

All analyses were performed in the R environment (version 3.6.1) or GraphPad Prism version 8.1. The average fluorescence intensity of the no-template control spots for a sample was subtracted from all fluorescence intensities for that sample prior to analysis to control for individual differences in seroreactivity to the IVTT reaction mixture.

Recovery of IgG from DBS relative to matched serum samples was estimated by least squares fitting (GraphPad Prism) to a model derived from the ligand-binding equation [30]:

1 $${S}_{DBS}= \frac{{S}_{max} \cdot r}{\frac{{S}_{max}}{{S}_{serum}}+r-1}$$

where *S*_*DBS*_ is the signal from a DBS sample, *S*_*serum*_ is the signal from the matched serum sample, *S*_*max*_ is the maximum signal, and r is the “recovery,” or the factor by which the antibody concentration in the DBS eluate is reduced relative to the serum concentration. Because the dilution of DBS eluates approximates that of a 1:100 serum dilution, the theoretical maximum recovery is 100%.

Background-subtracted fluorescence intensities were log2 transformed for group comparisons and regression analysis. A serorecognized antigen was defined as an antigen for which the average log2 transformed background-subtracted fluorescence intensity was significantly greater than that of the malaria-naïve North American control group (Mann-Whitney test, p < 0.05). Population seroreactivity was defined as the average seroreactivity of the population for antigens that were serorecognized.

To identify IgG markers for *P. falciparum* usPCR-positive infections, responses from *P. falciparum* usPCR-positive individuals were compared to usPCR-negative (including *P. vivax*-positive) individuals using a regularized logistic regression [31] performed with leave-one-out cross-validation. Prior to running the regression, input responses were narrowed to eliminate variables likely to primarily contribute noise. Specifically, the following filtering was performed: (1) antigens for which the signal standard deviation in endemic samples was less than or equal to that in the naïve North American controls were eliminated (36/249); (2) antigens with > 75% of antibody responses with signal below background were eliminated (7/213); and (3) a variance threshold based on the signal standard deviation (bottom 25% eliminated) was applied (52/206). The regularization parameter that minimized misclassification error was selected and used in the model. Informative antibody responses were identified as those with non-zero regression coefficients. Model performance was determined by receiving operating characteristic (ROC) analysis [32] on the cross-validated test predictions generated from all 225 patient samples with the optimized regularization parameter. The threshold for determining test characteristics of the model was obtained by Youden’s method (maximum distance from the line of identity) using the pROC package﻿.

### Data availability

All supporting data collected in this study are provided in Additional files 1 and 2.

## Results

### Participant demographics

The median age of participants was 35 years (range: 2–84), and 66% were female. Male participants were younger (median 17.5 years) than female participants (median 37.5 years). Despite the previous finding of *P. falciparum* and *P. vivax* prevalences of 8.7% and 5.6 %, respectively, by usPCR in 2015 [11], no participants in this study were positive for malaria by RDT or usPCR, and none reported recent malaria exposure. This lack of malaria infections and exposure was likely due to the relatively low number of enrolled males older than 20 years, who generally have a higher risk of occupational malaria exposure in Myanmar [10].

### Comparison of microarray signal from dried blood spots and serum

**Fig. 1:**
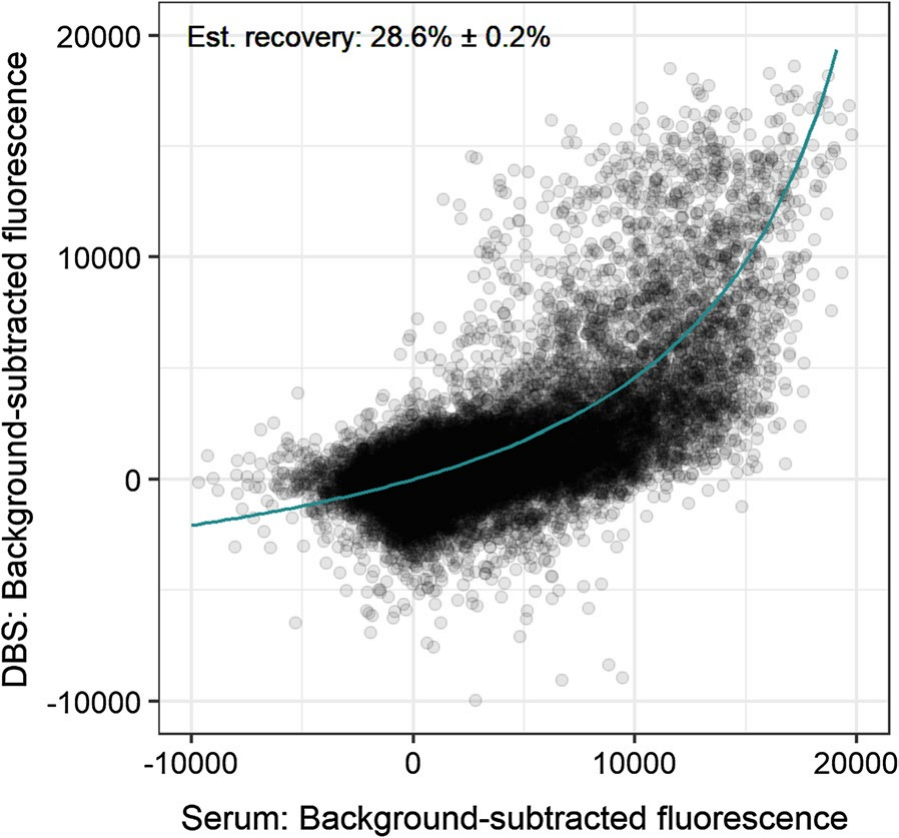
Comparison of Pf250 signal from DBS and serum. Line represents the best fit ligand binding-based model (Eq. 1) from which the estimated recovery parameter was derived. The error reported is the standard error in *r*

Matched DBS and serum samples were probed on protein microarrays populated with antigen fragments from the reference 3D7 *P. falciparum* genome. Figure 1 shows the relationship between the fluorescence intensities measured from DBS and those measured from serum. The overall recovery of IgG activity from DBS was estimated from a model derived from the ligand binding equation that treats the expected activity from DBS as a dilution of that from serum [30]. The model accounted for 46 % of the variance in the data, and approximately 28.6 % of the IgG activity measured in serum was observed from DBS.

### Antigen and population trends in dried blood spots and serum

**Fig. 2:**
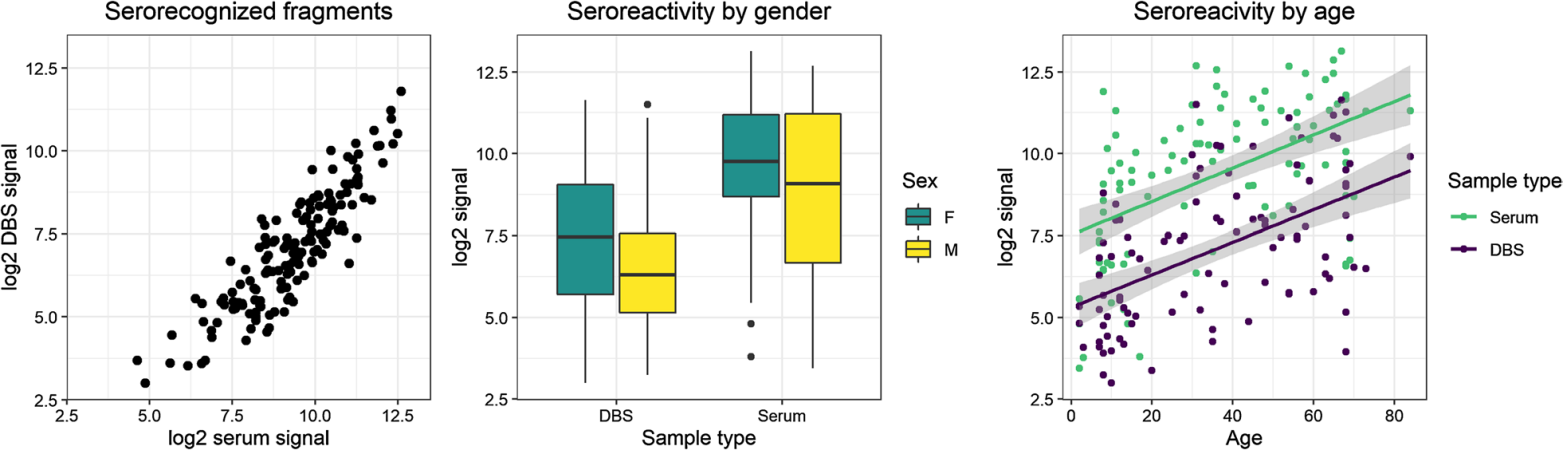
Antigen and population trends measured in dried blood spots (DBS) and serum. (Left) The average seroreactivity in DBS to fragments that were serorecognized in endemic samples was strongly correlated with that in serum (Spearman’s rho 0.8766, p < 0.0001). (Middle) Seroreactivity was higher on average for females in both DBS and serum. Note: points on boxplots are outliers as defined by the Tukey method. (Right) Although overall signal in DBS was lower than in serum, the increase in seroreactivity with age was consistent between the two sample types. Grey shading represents the 95% CI for the regressions. Spearman’s rho 0.5295 (p < 0.0001) and 0.5007 (p < 0.0001) for DBS and serum, respectively

Despite the loss of IgG activity with DBS, good agreement was seen in antigen and population trends between the two sample matrices. As expected, fewer fragments were serorecognized by the endemic groups in DBS eluates (156) compared to serum (231). However, 151 fragments were serorecognized from both DBS and serum, indicating high overlap between the two sample types. Moreover, the average seroreactivity against serorecognized fragments was strongly correlated between the two sample matrices (Spearman’s rho 0.8726, p < 0.0001) (Fig. 2, left). Population seroreactivity trends also agreed between the two sample types. The middle panel of Fig. 2 shows that females had an average higher seroreactivity to the serorecognized fragments than did males in both DBS and serum. This trend, consistent between the two sample matrices, is likely confounded by age, in that females were older and presumably had greater lifetime exposure to malaria. As expected, increasing seroreactivity was also observed with age with similar trends in serum (Spearman’s rho 0.5007, p < 0.001) and DBS (Spearman’s rho 0.5300, p < 0.001) despite lower signal from DBS (Fig. 2, right).

### Identifying IgG signatures of asymptomatic *P. falciparum* infection

To determine whether antibody responses could be useful markers of usPCR-positive *P. falciparum* infections, a case-control study was performed with 225 DBS collected as part of a previous study [11]. The baseline demographics for these groups are presented in Table 1.

**Table 1:** Baseline characteristics for case-control study

	Cases (Pf-pos) N = 50	Controls (Pf-neg) N = 175
Males	21 (42%)	87 (50%)
Age, years	19 (11–31)	24 (11–35)
Age category, years		
0–10	9 (18%)	38 (21%)
11–20	20 (40%)	29 (17%)
21–30	7 (14%)	41 (23%)
31–40	10 (20%)	37 (21%)
41+	4 (8%)	30 (17%)
Township		
Ann	20 (40%)	73 (42%)
Males	10 (50%)	47 (64%)
Age, years	23.5 (12.8–32.3)	30 (15–36)
Injangyang	30 (60%)	102 (58%)
Males	11 (37%)	40 (39%)
Age, years	14.5 (9.5–25)	22 (8–34.5)

Pf: *Plasmodium falciparum*

Categorical variables: n (%)

Continuous age: median (IQR)

**Fig. 3:**
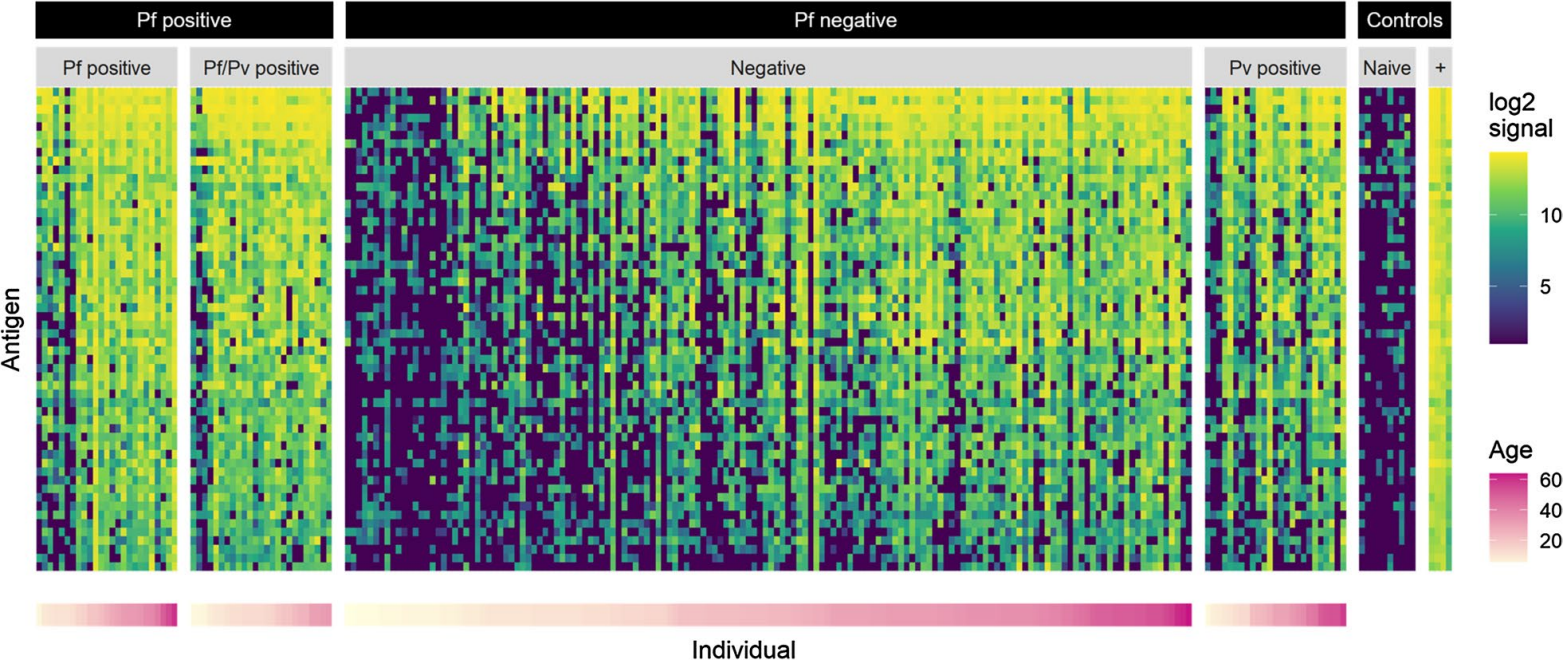
Heat map of seroreactivity against 56 differential antigen fragments (y-axis) on the Pf250 protein microarray. Pf positive group is shown on the left, Pf negative group in the middle, and assay controls on the right. Individuals (columns) are arranged according to increasing age within each group, and antigen fragments (rows) are arranged with descending mean log2 signal intensities

Using the same elution methodology for the matched DBS and serum study, antibodies were extracted from DBS and IgG activity was probed on a protein microarray populated with *P. falciparum* fragments from the 3D7 reference genome. In an initial analysis, 56 antigens were identified that were serorecognized by the *P. falciparum*-positive group and signal intensity differed between the case and control groups (Mann-Whitney test, p < 0.05). The log2-transformed fluorescent signal from all individuals against the differential antigens are shown in Fig. 3. In all groups, older individuals had greater intensity and breadth of seroreactivity compared to younger individuals.

**Fig. 4:**
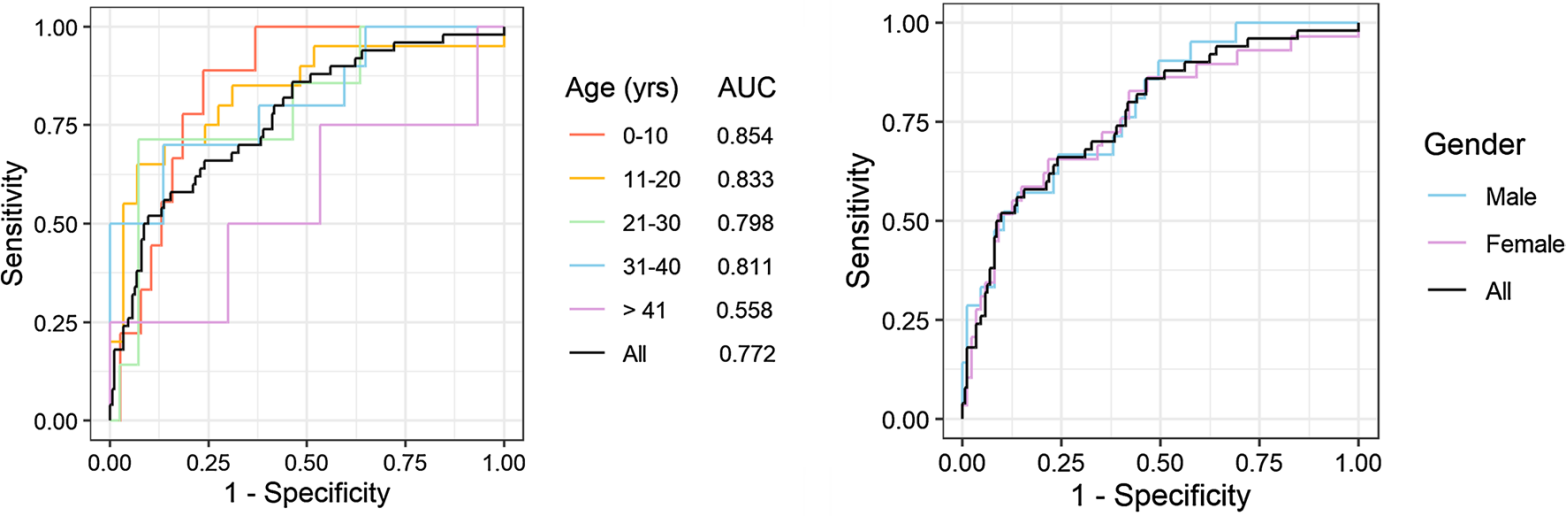
ROC curves for 18 antibody responses predictive of Pf-positivity by usPCR. Model performance is affected by age (left) but not gender (right). AUC: area under the curve; ROC: receiving operator characteristic; Pf: *Plasmodium falciparum*; usPCR: ultrasensitive PCR

A regularized logistic regression model was then fit using leave-one-out cross-validation to identify which IgG responses may be useful for classifying cases and controls. Table 2 lists the 18 antigens for which model coefficients were non-zero, indicating that those IgG responses affected the odds of an individual being usPCR-positive for *P. falciparum*. Notably, the most influential antibody response was that against *P. falciparum* circumsporozoite protein (CSP), which had a strong negative impact on the odds of *P. falciparum*-positivity. Figure 4 shows the overall performance of the logistic regression model in predicting *P. falciparum*-positivity, broken down by age and gender. While there was no noticeable difference in model performance for males and females, age had a significant impact on how well the model performed. IgG responses were better predictors for younger individuals and were not predictive of *P. falciparum*-positivity in older adults (> 41). Across all individuals included in the study, model area under the curve (AUC) of the ROC was 0.772 and performance characteristics were 58% sensitivity, 85% specificity, 88% negative predictive value, and 52% positive predictive value, relative to usPCR.

**Table 2:** Informative antibody signatures of low-density *P. falciparum* infections

Rank	Direction^a^	Gene identification	Description
1	–	PF3D7_0304600	Circumsporozoite protein (CSP)
2	+	PF3D7_1149600	DnaJ protein, putative
3	+	PF3D7_0407700	Conserved Plasmodium protein
4	+	PF3D7_1420700	Surface protein P113
5	+	PF3D7_0903500	Nucleoporin NUP138, putative
6	+	PF3D7_0223300	Erythrocyte membrane protein 1 (PfEMP1), exon 2
7	+	PF3D7_1250600	Translation initiation factor eIF-2B subunit beta, putative
8	+	PF3D7_1035600	Merozoite surface protein (MSP)
9	+	PF3D7_1221900	Conserved Plasmodium membrane protein
10	+	PF3D7_1211900	Non-SERCA-type Ca2+ -transporting P-ATPase
11	–	PF3D7_1149200	Ring-infected erythrocyte surface antigen, RESA3
12	+	PF3D7_0801000	Plasmodium exported protein (PHISTc)
13	+	PF3D7_1036000	Merozoite surface protein 11
14	+	PF3D7_1433500	DNA topoisomerase 2
15	+	PF3D7_0404600	Conserved Plasmodium membrane protein
16	+	PF3D7_0402400	Plasmodium exported protein, GEXP18
17	–	PF3D7_1036400	Liver stage antigen 1 (LSA1)
18	+	PF3D7_0827600	Conserved Plasmodium protein, unknown function

^a^ + indicates a positive association with Pf-positivity

## Discussion

This study demonstrated that DBS are viable samples for probing IgG responses on malaria protein microarrays. Despite a loss of signal relative to serum, IgG activity from DBS was successfully quantified, and similar trends were seen for antibodies derived from DBS and serum. Antibodies eluted from DBS were also found to be useful for identifying informative IgG responses in individuals who have asymptomatic, subpatent *P. falciparum* malaria.

Matched DBS and serum samples were probed on Pf250 protein microarrays, and approximately 30% of the IgG activity in serum was recovered from DBS. This recovery was lower than expected, as ELISA-based methods have reported up to 70% recovery of malaria IgG [30], and the DBS elution procedure was designed to approximate the 1:100 serum dilution in the standard Pf250 protocol. It is unlikely that sample degradation played a role in this loss of IgG activity from DBS, as the DBS and serum were placed in the freezer at the same time, a strength of this study. As such, the loss of IgG activity can likely be attributed to the comparatively gentle, overnight DBS elution protocol. Many elution methods exist, and while harsh conditions may decrease antibody activity by denaturing or shearing antibodies, gentler methods such as the one used in this study may not completely elute all of the available antibodies. Additional optimization may identify ideal conditions for maximizing elution while minimizing antibody denaturation.

Although recovery of IgG activity from DBS was lower than expected, similar antigen and population trends were observed for antibodies obtained from both sample matrices. There was a large overlap of serorecognized antigens, with over 65% of the fragments that were recognized in serum also being serorecognized in DBS eluates. Average antibody response signal against serorecognized fragments was strongly correlated between the two sample matrices, indicating that DBS eluates provided reliable quantitative results compared to serum. Similarly, despite the overall lower signal from DBS, population trends in seroreactivity by gender and by age were comparable between the two sample matrices. Thus, IgG activity measured on protein microarrays from DBS can be relied on for observing antigen and population trends in malaria-endemic areas. These results likely extend to infections beyond malaria for which serosurveillance is routinely used to track exposure or vaccination coverage, and DBS collection would be more logistically feasible and cost effective than serum collection for serosurveillance of a number of infectious pathogens.

The case-control study aimed to identify serological signatures of asymptomatic *P. falciparum* infections. Previously collected and banked DBS were eluted using the same method as the serum/DBS comparison study and probed on Pf250 microarrays. While the study was designed to identify markers of asymptomatic infection, it is important to note that serological profiles also reflect past malaria exposure, which is likely associated with infection status. Among the 18 antibody responses that affected the odds of usPCR positivity, 15 were positively correlated and three were negatively correlated with *P. falciparum* infection. The antibody response with the greatest magnitude of negative or positive association with asymptomatic infection was to CSP, a leading malaria vaccine candidate [33]. In this study, IgG activity against CSP was negatively correlated with *P. falciparum*-positivity, consistent with CSP antibodies having a protective effect against infection as previous studies have reported [34, 35]. Many of the other antibody responses identified here have also been identified in other studies as markers of *P. falciparum* exposure or asymptomatic infection using similar protein microarray methods with serum samples [14, 17, 18, 36, 37]. For example, five of the 18 antibody responses identified here were also implicated by Helb et al. as predictors of the number of days since previous *P. falciparum* infection or of infection incidence in the last year [14]. These consistent findings between the present study and previous work provide further evidence that DBS are useful samples for profiling *P. falciparum*-specific immune responses on protein microarrays.

The antibody responses against the 18 antigens in Table 2 could be combined to classify individuals as usPCR-positive and usPCR-negative with modest performance (AUC = 0.772). Model performance was inversely related to age, working better in younger age groups (0–10, AUC = 0.854) compared to older age groups (41+, AUC = 0.558). This age effect is likely due to repeated malaria exposure contributing to increased breadth and intensity of malaria-specific circulating antibodies in older people that obfuscated the signatures attributable to usPCR-positivity.

The test characteristics of the model relative to usPCR were modest across all samples, with 58% sensitivity, 85% specificity, 88% NPV, and 52% PPV, suggesting that these serological signatures alone would not be sufficient for accurately identifying usPCR-positive individuals. However, it is important to note that all of the individuals included in this case-control study were RDT-negative. While the model does not accurately capture all usPCR-positive infections, it does capture more of the asymptomatic reservoir than standard RDTs currently used for malaria diagnosis, though at the cost of reduced specificity, particularly for adults or individuals with a history of previous exposure. Given this improved sensitivity over RDTs, serological markers could be useful intermediate tools for identifying exposure to ultra-low density malaria infections, particularly if serosurveillance strategies target individuals with less previous exposure, such as children, as sentinels for recent population exposure.

## Conclusions

Screening malaria-specific antibody responses on protein microarrays can help identify immune factors that elicit protection from malaria infection, disease, and transmission, as well as for markers of past exposure. Most malaria protein microarray work has relied on serum as a sample matrix, which requires immediate laboratory processing and continuous cold chain. This study demonstrated that malaria-specific immune responses can be detected, quantified, and analysed from DBS on protein microarrays. As a proof-of-concept, DBS from asymptomatic *P. falciparum* cases and controls were probed to identify a panel of antibody responses associated with increased odds of asymptomatic malaria infection. The use of DBS on protein microarrays enables high-dimensional serological studies in populations where serum collection, transport, and storage would be otherwise impossible.

## Supplementary Information

**Additional file 1. **Sample metadata and Pf250 signal for matched DBS and serum study.

**Additional file 2. **Sample metadata and Pf250 signal for case-control study.

## Data Availability

The datasets generated and analysed during this study are included in this published article and its additional files.

## Abbreviations

DBS: Dried blood spot
usPCR: Ultrasensitive polymerase chain reaction
RDT: Rapid diagnostic test
Pf250: Protein microarray populated with *Plasmodium falciparum* antigen fragments
RT-PCR: Reverse transcription polymerase chain reaction
IVTT: In vitro transcription and translation
ROC: Receiver operator characteristic
Pf: *Plasmodium falciparum*
CSP: Circumsporozoite protein
AUC: Area under the curve
